# Med-ImageTools: An open-source Python package for robust data processing pipelines and curating medical imaging data

**DOI:** 10.12688/f1000research.127142.3

**Published:** 2025-02-07

**Authors:** Sejin Kim, Michal Kazmierski, Kevin Qu, Jacob Peoples, Minoru Nakano, Vishwesh Ramanathan, Joseph Marsilla, Mattea Welch, Amber Simpson, Benjamin Haibe-Kains

**Affiliations:** 1 Princess Margaret Cancer Centre, University Health Network, Canada, Toronto, ON, Canada; 2 Department of Medical Biophysics, University of Toronto, Toronto, ON, Canada; 3 Faculty of Engineering, University of Toronto, Toronto, ON, Canada; 4 Centre for Health Innovation, Queen's University and Kingston Health Science Centre, Kingston, ON, Canada; 5 Vector Institute, Toronto, ON, Canada

**Keywords:** medical imaging, deep learning, open source, data processing, dicom, nifti, nnunet

## Abstract

**Background:**

Machine learning and AI promise to revolutionize the way we leverage medical imaging data for improving care but require large datasets to train computational models that can be implemented in clinical practice. However, processing large and complex medical imaging datasets remains an open challenge.

**Methods:**

To address this issue, we developed Med-ImageTools, a new Python open-source software package to automate data curation and processing while allowing researchers to share their data processing configurations more easily, lowering the barrier for other researchers to reproduce published works.

**Use cases:**

We have demonstrated the efficiency of Med-ImageTools across three different datasets, resulting in significantly reduced processing times.

**Conclusions:**

The AutoPipeline feature will improve the accessibility of raw clinical datasets on public archives, such as the Cancer Imaging Archive (TCIA), the largest public repository of cancer imaging, allowing machine learning researchers to process analysis-ready formats without requiring deep domain knowledge.

## Introduction

Radiology is a powerful modality of data for clinical work — it gives clinicians the ability to see the inner workings of the human body, that cannot be seen from the outside. ^1^ They can inspect in 2D or 3D, the anatomy surrounding the disease, enabling key information to make life-altering clinical decisions. While regular images taken on cameras or phones are stored in a variety of accessible formats, medical images are encoded in the Digital Imaging and Communications in Medicine (DICOM) standard file format. ^2^

The DICOM standard was developed in the 1980s due to the increasing need for an interoperable standard for 3D medical images across various manufacturers. ^2^ A key feature of DICOM is the plethora of metadata fields that store information beyond imaging data, such as patient information, clinical variables, and acquisition parameters. As modern medical practice evolved over the years, the DICOM standard has grown to accommodate more metadata fields and encompass new imaging modalities or therapy information. ^2^ This type of data format is unsuitable for imaging analysis as the relevant voxel array must be manually accessed through DICOM hierarchy. ^2^ Furthermore, 3D scans are acquired on a slice-by-slice basis; thus, researchers must stitch together data from multiple files to create one 3D image, adding delays caused by disk reads and consolidation processes. ^2^

Specialties that rigorously use imaging data are heavily reliant on DICOM, one of which is radiation oncology. Images are used along every step of the clinical workflow: from deriving a precise diagnosis, to designing personalized radiation therapy plans, and delivering each radiation dose with the appropriate alignment and orientation with brief scans. While the defined standard serves as a good guideline, each manufacturer has slightly different implementations. This is especially the case for DICOM-RT (radiotherapy), a subset of modalities for communicating radiotherapy data. ^2^ The DICOM-RT standard includes additional modalities such as RTStruct for contour data, and RTDose for radiotherapy dose maps and dose-volume histograms (DVH). ^2^ While the broad adoption of the DICOM standard to accommodate for various use cases has allowed it to become the defacto standard for encoding, storing, and transferring of medical images, its comprehensive nature has made it difficult for researchers to navigate for the purposes of imaging projects. ^2^

## Current workflows

The Cancer Imaging Archive (TCIA) (RRID:SCR_008927) is one of the largest public repositories of DICOM images available, with over 140 datasets consisting of more than 60,000 patients. ^3^ The datasets undergo a quality assurance process to ensure the recorded clinical variables are coherent and the DICOM files are not missing any important metadata fields. ^3^ These stringent processes and infrastructure have allowed TCIA to become one of the most comprehensive repositories for biomedical imaging datasets, inviting researchers from different fields to explore new ideas and methods on high quality datasets. ^3^

While the underlying data and its annotations are of clinical quality, processing the dataset for subsequent analysis requires a non-trivial amount of effort: manually reorganizing directories and matching radiation therapy structures (RTStruct), referred to as DICOM-RT contours, and radiation therapy plans (RTDose/RTPlan) to its corresponding images. ^4^ This is partly due to the inherently complex nature of clinical datasets, as data is collected on the basis of need and iterative improvements, not structured scientific inquiries. It is also sometimes due to the lack of familiarity from machine learning (ML) and artificial intelligence (AI) researchers in handling the DICOM files for their analytical pipelines. Typical AI imaging datasets have pairwise associations of one image to a single ground-truth label. ^5^ However, one patient in clinical datasets may have multiple RTStruct and RTDose files with one imaging acquisition, one RTStruct and RTDose with multiple images, or worst of all, multiple RTStruct and RTDose files with multiple images. ^6^ In any of these cases, the directories are not always intuitively structured to help researchers understand which files correspond with another.

Once researchers have successfully curated the dataset into an organized structure for analysis, in order to process the raw dataset into analysis-ready format, they must choose from a variety of processing parameters, ranging from voxel spacing, RTStruct name parsing, and hounsfield unit (HU) window levels, based on the design of their analysis. While these implicit decisions for image processing are often arbitrary, they can greatly impact model training and performance, but are not transparently disclosed in publications. ^7^ This leads to the difficulty of reproducibility of medical deep learning research, adding another deterrence to clinical adoption.

Furthermore, there are a limited number of software packages that researchers can use to quickly parse DICOM-RT files into analysis-ready arrays ( Table 1). Chief among those, SlicerRT, ^8^ an extension of 3Dslicer ^9^ (RRID:SCR_005619), an open-source DICOM visualization tool, has been widely used by the medical imaging community. Despite its broad adoption, batch data processing with Slicer requires custom scripting in Python, to be executed in the Slicer ecosystem. Rather than simply installing a package within their Python environment, users must install the Slicer application and add any other dependencies, not provided by Slicer, into the application environment. As a result, machine learning projects relying on Python for data preprocessing will have their code fragmented across multiple environments–the Slicer environment for data processing, and another Python environment for data analysis and machine learning. ^10^ RT-Utils is a lightweight, open-source Python package designed to handle RTStruct files with relative ease and simplicity, allowing users to easily export contours into segmentation masks in arrays. However, the functionalities of RT-Utils are limited to the RTStruct modality. PlatiPy is a recent processing library and analysis toolkit for medical imaging, mainly designed for the context of radiation therapy. It features a comprehensive set of image manipulation functions such as registration and atlas-based segmentation methods, allowing researchers the flexibility to process imaging data into any format they need. However, PlatiPy does not solve the inherent complexity of clinical datasets, and researchers must spend hours reorganizing the data into a structured set of samples and labels. The current landscape of open-source medical imaging tools highlights the need for a native Python package that can parse large DICOM/DICOM-RT datasets to an analysis-ready format for ML/AI development in a consistent, reproducible workflow.

**Table 1.** Comparison of existing medical imaging processing packages and their features. RTSTRUCTs: DICOM-RT Contours; RTDOSEs: DICOM-RT Dose; CT: Computed Tomography; MRI: Magnetic Resonance Imaging; NifTI: Neuroimaging Informatics Technology Initiative; Nrrd: Nearly raw raster data; DICOM: Digital Imaging and Communications in Medicine.

	3Dslicer + SlicerRT	RT-Utils	PlatiPy	Med-ImageTools
Native Python interface		⬤	⬤	⬤
Command-line interface				⬤
Handles RTSTRUCTs	⬤	⬤	⬤	⬤
Handles RTDOSEs	⬤		⬤	⬤
Handles images (CT/MRI)	⬤		⬤	⬤
Built-in image transformations	⬤		⬤	⬤
Exports to analysis-ready NifTI/Nrrd	⬤		⬤	⬤
Image registration	⬤		⬤	
Built-in bulk processing of entire datasets				⬤
Automatic parsing of DICOM metadata	⬤			⬤

To address the limitations of the current software packages used to process medical images, we developed Med-ImageTools, ^11^ a new Python package designed to help researchers transform complex medical datasets into analysis-ready format with few lines of code. It is also focused on helping researchers develop transparent and reproducible medical image processing pipelines by addressing most of the boilerplate code required for image transformations and processing parallelization. While Med-ImageTools has many modular functions for image, contour, and dose input/output (IO) built on popular frameworks such as SimpleITK, TorchIO and PyDicom, such functionalities are redundant and available in other open-source packages as well. We have tailored our core features towards broader use cases and development workflows, instead of modality or disease type specific workflows, such as BIDS. ^12^ Our main contribution is in the development of AutoPipeline and will mainly discuss its functionalities and implementation.

## Methods

### AutoPipeline

**Figure 1.**
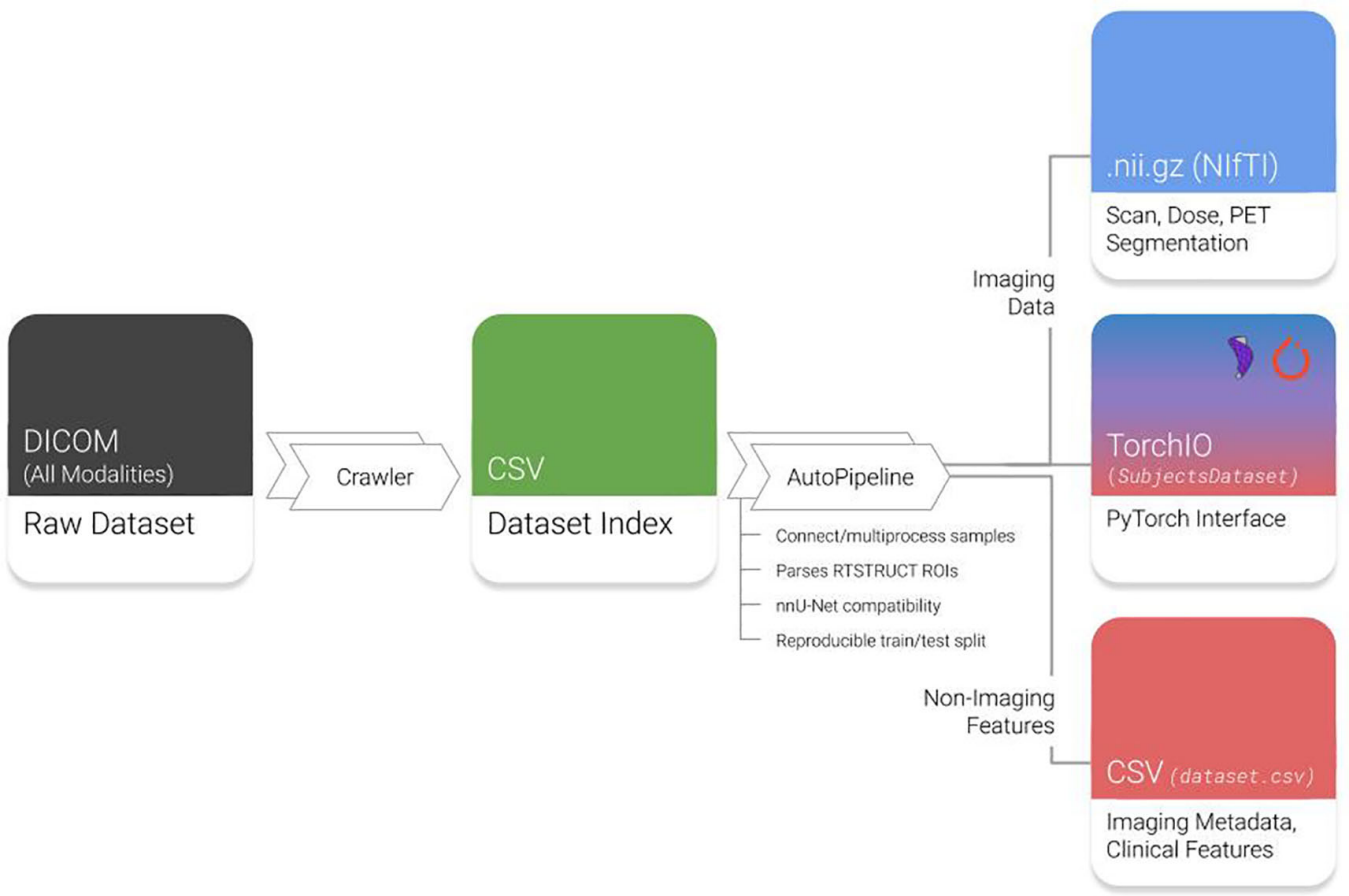
Overview of Med-ImageTools features. Raw datasets are indexed by the Crawler module and automatically processed by AutoPipeline. DICOM: Digital Imaging and Communications in Medicine; RTSTRUCTs: DICOM-RT Contours; PET: Positron emission tomography. NifTI: Neuroimaging Informatics Technology Initiative; CSV: Comma-separated values.

AutoPipeline is the main feature of Med-ImageTools that allows users to easily process raw DICOM clinical datasets into analysis-ready Nrrd or NifTI files, which are commonly used file formats for 3D volumetric data. It is interfaced using the command line, so the user only needs to submit a single command into the terminal to execute the three core steps in the AutoPipeline process ( Figure 1):

1. **Crawl**: The crawler opens every DICOM file in the dataset using Pydicom, indexing important metadata, such as unique identifiers, modality information, and references to other modalities. This produces a database of every unique image and DICOM-RT modality.
2. **Connect**: In this step, each patient’s indices of unique files of different modalities are connected to form one coherent sample. There are various heuristics the user may choose to connect samples. The default option is through DICOM metadata, as datasets derived from clinical practice are expected to have corresponding metadata that references unique identifiers of the parent image or RTPlan. Alternative heuristics allow users to deal with anomalies with corrupted or missing metadata.
3. **Process**: All identified samples are processed according to imaging and transformation parameters defined by the user. The user can configure parameters such as the pixel spacing in mm(s), which specific modalities to process, the number of cores they want to use for multiprocessing, and define nnU-Net specific flags as well. The images are manipulated using SimpleITK without requiring any user intervention.

The AutoPipeline feature can be directly interfaced once Med-ImageTools is installed. To install Med-ImageTools, the recommended method is *via* the PyPI package repository in a virtual environment. Running the ‘ *pip install med-imagetools *’ command will install the latest version of Med-ImageTools and its associated dependencies defined by the requirements.txt file. Now, whenever the user is in the virtual environment where Med-ImageTools is installed, they can directly interact with the AutoPipeline feature in the command line. The simplest way to use it is through ‘ *autopipeline input_directory output_directory*’. This will automatically process the dataset located in ‘ *input_directory*’ and process them at ‘ *output_directory*’ using the default parameters. Med-ImageTools will generate files that are not analysis-ready images, such as the autogenerated ‘Dataset Index’ ( Figure 1), and store them in a folder named “.imgtools” at the path of the ‘input_directory’. This is to ensure a convenient user experience and intuitive folder structure by hiding extraneous components. An extended tutorial of AutoPipeline and all its associated parameters are available here. At the present time, there are no minimum system requirements for Med-ImageTools as it will run regardless of number of processor cores or memory (RAM). However, if the researcher can leverage greater number of cores and RAM, it will allow the AutoPipeline to be parallelized and process the data faster.

As the crawl and process steps are computationally intensive, all steps in AutoPipeline are automatically parallelized using the joblib backend to efficiently leverage all available computational resources. While the output of the AutoPipeline processing can result in terabytes of images, the crawl is limited to a few kilobytes of a metadata database, making it an ideal asset to share with other researchers as a detailed descriptor of a medical imaging dataset. We therefore propose to attach the crawled metadata spreadsheet to large TCIA datasets to allow Med-ImageTools users to process large datasets much faster and more efficiently. These databases are expected to save up to 1000 core-hours of crawling per dataset, accumulating over 2000 core-hours of computation saved per user. By standardizing a commonly repeated imaging processing pipeline into a single unified package, we hope to improve the reproducibility and transparency of future medical imaging research.

## Use cases

We showcase the value of the AutoPipeline implemented in Med-ImageTools v1.0.0 for processing three medical imaging datasets, namely Pancreatic-CBCT-SEG from TCIA, liver metastasis private dataset and RADCURE pending public release on TCIA, in order of complexity and sizes. In each use case, we initially describe the process.

### Using pre-crawled datasets on the Pancreatic Cone-beam Computed Tomography (CBCT) ^13^

*40 patients with abdominal CBCT scans and their associated contours of regions of interest and other organs, publicly available on TCIA*

**Figure 2.**
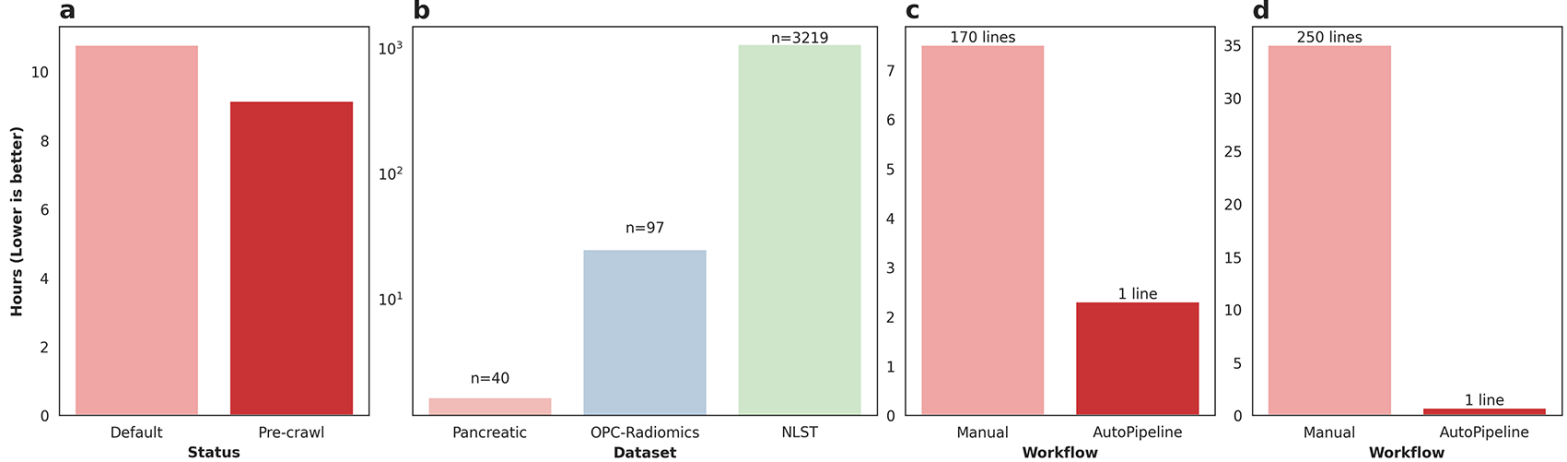
a) Time taken to process the Pancreatic-CT-CBCT-SEG dataset with (Pre-crawl) and without (Default) the pre-crawled databases. b) Amount of core-hours saved by using pre-crawled databases across various public TCIA datasets. c) Time taken to process the liver metastasis dataset manually vs AutoPipeline. d) Time taken to process the RADCURE dataset manually vs AutoPipeline.

The Pancreatic-CT-CBCT-SEG dataset was processed twice using AutoPipeline: once from scratch, and once using the pre-crawled dataset, available on the tcia-crawls branch of Med-ImageTools. Processing from scratch, the dataset took 10.77 core-hours (10:46), whereas using a pre-crawled database allowed the processing to finish in 9.14 core-hours (9:08) ( Figure 2a). The pre-crawled database reduced processing time by 1.63 core-hours, representing an 18% increase in total processing speed or 2 minutes 27 seconds per patient. The time saved from pre-crawled databases is not a substantial quantity for datasets with less than 100 patients. However, when scaled up to larger TCIA datasets such as OPC-Radiomics ^14^ (n=606) and NLST ^15^ (n=26254), it can save researchers 24.7 core-hours and 1072 core-hours, respectively ( Figure 2b). The resources saved are reported in core-hours to allow a hardware-agnostic estimation of time and cost savings. While it may vary depending on the research infrastructure utilized, these databases can result in significant savings in billing.

### Comparing manual vs automatic processing of the liver metastasis dataset

*97 patients with abdominal CT scans and their associated contours of liver and gross tumour volumes (GTV), data access described in data availability section below.*

The liver metastasis dataset was processed using the Slicer API to export a CT scan along with segmentations of the liver and GTVs for each patient. In the initial DICOM dataset, each patient had a single RTSTRUCT file along with one or more CT series, one of which was referenced by the RTSTRUCT. The first step of the process was to load the entire DICOM dataset into the Slicer DICOM database *via* the graphical user interface (GUI), to make the data available inside the Slicer scripting environment. Then, we wrote a script to export an initial set of candidate segmentations for the liver and GTVs for each patient, along with the referenced CT scan. This script leveraged the Slicer API to identify the CT series that was referenced by the patient’s RTSTRUCT and used an *ad hoc* string filtering function to identify candidate segmentations. Finally, we iteratively refined the set of exported RTSTRUCT contours on a patient-by-patient basis, based on visual checking, and physician feedback. Although this step was time-consuming, there is, at present, no substitute for manual verification to ensure data quality and correctness. On the other hand, the initial database construction and export script using the Slicer API achieved results roughly analogous to the automatic output of AutoPipeline. The export script is approximately 170 lines long and took 7.5 hours to run on the whole dataset-this script is available on our GitHub repository. In contrast, on the same 6-core machine (16GB RAM, Windows 10), AutoPipeline took only 2.3 hours with 1 command line, mainly accelerated by parallelization ( Figure 2c). The authors involved in validating Med-ImageTools’ effectiveness on this private dataset had no active involvement in the development of the package before its application. This highlights the package’s robustness on unseen data and its potential utility for multi-centre collaborations to ensure consistent processing. One use case might be to enable federated learning platforms to automatically process each node’s datasets without requiring any user intervention.

### Comparing manual vs automatic processing of the RADCURE dataset

*3,219 patients with head and neck CT scans and their associated contours of organs at risk (OAR) for radiotherapy, pending public release on TCIA.*

The RADCURE dataset is a large dataset of 3,219 head and neck cancer patients and their radiotherapy planning data. The dataset was extracted from two separate treatment planning software systems, meaning the directories and DICOM metadata were structured differently. The directories from each system were restructured using general heuristics, and any abnormal cases were flagged and manually organized. We used SimpleITK and PyDicom to extract the imaging and contour data from the DICOM files, which underwent a similar iterative process as the liver metastatic dataset. The script is over 1000 lines long and takes 30-40 hours to run on the whole dataset using a single core-this script is available on our GitHub repository. AutoPipeline scales dramatically based on the number of cores available, which enables the entire dataset to be processed in 40 minutes using 32 cores, automatically managing the directories from different systems and extracting the contours ( Figure 2d). These results demonstrate Med-ImageTools’ design does not bottleneck any multiprocessing backends and brings meaningful acceleration on very large datasets, such as RADCURE.

One caveat of these comparisons is that the iterative process of filtering appropriate contours, which can add weeks to months of cooperation between the researchers and the physicians, were already conducted in the original processing steps. Various data cleaning steps that require human intervention, such as sorting contour names or selecting specific subseries acquisitions, cannot be fully automated for the foreseeable future. The Med-ImageTools team aims to add features to the package that will assist researchers in these steps, such as adding a flag to visualize all unique contours without requiring code, adding subseries detection to the crawler, and publishing a set of regular expressions (regex) that can be used to automatically choose contours from prominent head and neck datasets on TCIA. Also, these comparisons do not take into account the time it takes for researchers to develop the manual processing scripts. Hence, the actual amount of time saved for the researcher may be greater than the reported times.

## Discussion

Although ML and AI promise to revolutionize the way we leverage medical imaging data for improving care, they require large datasets to train computational models that can be implemented in clinical practice. However, processing large and complex medical imaging datasets remains an open challenge. To address this issue, we developed Med-ImageTools, a new open-source software package to automate data curation and processing while allowing researchers to share their data processing configurations more easily, lowering the barrier for other researchers to reproduce published works.

The AutoPipeline feature will improve the accessibility of raw clinical datasets on public archives, such as TCIA, allowing machine learning researchers to process analysis-ready formats without requiring deep domain knowledge. Another exciting potential of Med-ImageTools lies in building automated workflows using AutoPipeline. For a researcher to build an end-to-end automated pipeline starting from clinical DICOM datasets to outputting an inference-ready deep learning model, they could easily develop a reproducible processing step by configuring only the command line interaction of Med-ImageTools, making debugging and custom configurations simpler since the developer would not have to rely on a static script.

While our package aims to address challenges encountered across a few medical imaging labs, we acknowledge that there may be infinite other issues that may arise in DICOM datasets. This is one of the key reasons why our package is open-source for community involvement and contribution. Also, as stated previously, there are certain onerous tasks that cannot be automated and must undergo human supervision. These aspects of researcher-clinician collaboration are an inevitable part of medical imaging research and are subject to delay.

No single solution can completely solve the reproducibility crisis of medical deep learning research, due to a variety of issues ranging from ambiguous data processing techniques to stochasticity of model training. However, community-centered open-source solutions and increased clinical adherence to data standards, such as contour nomenclature, ^16^ can incrementally improve research quality and reproducibility, and make medical deep learning research more accessible for everyone.

## Data Availability

The Pancreatic-CT-CBCT-SEG dataset is available on The Cancer Imaging Archive at: https://doi.org/10.7937/TCIA.ESHQ-4D90. The pre-crawled Med-ImageTools database of the Pancreatic-CT-CBCT-SEG dataset is available on GitHub at: https://github.com/bhklab/med-imagetools/raw/tcia-crawls/csvs/imgtools_Pancreatic-CT-CBCT-SEG.csv. The liver dataset was collected as part of the study titled *Radiomics artificial intelligence modelling for prediction of local control for colorectal liver metastases treated with radiotherapy* ^17^ with Institutional Review Board and informed consent. Interested individuals should contact the senior author for access to the dataset, which will be made available following standard institutional rules for IRB and HIPAA. The RADCURE dataset is pending public release on TCIA and the article will be updated upon release. Data published on TCIA are subject to TCIA data usage policies and restrictions.

## Software availability

Source code available from: github.com/bhklab/med-imagetools also available on the PyPI software repository: pypi.org/project/med-imagetools.

Scripts for use cases are available from: github.com/bhklab/med-imagetools/tree/F1000Research

Archived source code at time of publication: https://doi.org/10.5281/zenodo.14766792 ^18^

License: MIT

Its associated documentation and tutorials are available at: https://bhklab.github.io/med-imagetools
