# Supplementary material for: Med-ImageTools: An open-source Python package for robust data processing pipelines and curating medical imaging data
Source: F1000Res. 2025 Feb 7;12:118. Originally published 2023 Feb 1. [Version 3] doi: 10.12688/f1000research.127142.3 (PMC11847151; doi:10.12688/f1000research.127142.3)
Supplement: Peer review history

# Reviewer report: Reviewer response for version 3

**DOI:** 10.5256/f1000research.177718.r366375

**Published:** 2025-02-22

**Authors:** Johann Faouzi

**Affiliations:** 1 ENSAI, Rennes, France

I would like to thank the authors for their detailed response. The software development aspects have been substantially improved since the previous version, and I am happy to approve the current version of the article.

Are the conclusions about the tool and its performance adequately supported by the findings presented in the article?

Yes

Is the rationale for developing the new software tool clearly explained?

Yes

Is the description of the software tool technically sound?

Yes

Are sufficient details of the code, methods and analysis (if applicable) provided to allow replication of the software development and its use by others?

Yes

Is sufficient information provided to allow interpretation of the expected output datasets and any results generated using the tool?

Yes

Reviewer Expertise:

Open source software, Python programming, machine learning, medical applications

I confirm that I have read this submission and believe that I have an appropriate level of expertise to confirm that it is of an acceptable scientific standard.

# Reviewer report: Reviewer response for version 2

**DOI:** 10.5256/f1000research.164713.r274815

**Published:** 2024-06-12

**Authors:** Rachel Sparks

**Affiliations:** 1 King's College London, London, England, UK

I am happy with the revisions made to the document and responses to my specific queries.

Are the conclusions about the tool and its performance adequately supported by the findings presented in the article?

Yes

Is the rationale for developing the new software tool clearly explained?

Yes

Is the description of the software tool technically sound?

No

Are sufficient details of the code, methods and analysis (if applicable) provided to allow replication of the software development and its use by others?

No

Is sufficient information provided to allow interpretation of the expected output datasets and any results generated using the tool?

No

Reviewer Expertise:

Software development; open source software; medical image computing

I confirm that I have read this submission and believe that I have an appropriate level of expertise to confirm that it is of an acceptable scientific standard.

# Reviewer report: Reviewer response for version 2

**DOI:** 10.5256/f1000research.164713.r274816

**Published:** 2024-05-15

**Authors:** Johann Faouzi

**Affiliations:** 1 ENSAI, Rennes, France

I would like to thank the authors for replying to all my comments. I think that the manuscript has been improved since the first version.

 Nonetheless, I still have some concerns regarding the source code. Despite some cleaning, there are still numerous flake8 errors. Flake8 is not used in the CI even though it is installed. Code coverage is still not included in the CI. PR with failing CI are still merged into in the main branch. Overall, the software aspect of this contribution, which is the main contribution, has still important limitations.

Are the conclusions about the tool and its performance adequately supported by the findings presented in the article?

Yes

Is the rationale for developing the new software tool clearly explained?

Yes

Is the description of the software tool technically sound?

Yes

Are sufficient details of the code, methods and analysis (if applicable) provided to allow replication of the software development and its use by others?

Yes

Is sufficient information provided to allow interpretation of the expected output datasets and any results generated using the tool?

Yes

Reviewer Expertise:

Open source software, Python programming, machine learning, medical applications

I confirm that I have read this submission and believe that I have an appropriate level of expertise to confirm that it is of an acceptable scientific standard, however I have significant reservations, as outlined above.

# Response

**Published:** 2024-05-29

**Authors:** Benjamin Haibe-Kains

**Affiliations:** Princess Margaret Cancer Centre, Canada

Thank you Dr Faouzi for your feedback regarding our CI/CD pipeline and we are glad to inform you that we have made the appropriate updates. 

 We have switched from flake8 to ruff (Rust-based Python linter) as our main linter and have made appropriate modifications to our codebase to address all linting issues. The linter is also integrated into the CI/CD pipeline as well. 

 The main failure of our previous tests was due to the deprecation of .egg uploads to PyPI, despite the unit tests always passing. We have resolved these issues by rewriting our CI/CD pipeline, which has led to reliable tests and continuous deployments to PyPI. 

 We have also incorporated code coverage into our CI/CD pipeline and have added a badge to the README. While the current code coverage of the tests is still low, we believe after our upcoming refactoring efforts, the coverage of the tests will significantly improve. 

 Finally, we have implemented semantic versioning to our package to adhere to industry standards and ensure consistent, trackable versioning changes.

# Reviewer report: Reviewer response for version 1

**DOI:** 10.5256/f1000research.139617.r189038

**Published:** 2023-10-17

**Authors:** Johann Faouzi

**Affiliations:** 1 ENSAI, Rennes, France

The authors present their Python package for data processing pipelines and curating medical imaging data in this manuscript. Overall the manuscript is well written and clearly presents the main contributions of this package to the community. Nonetheless, some points on the software side could be improved. Please find my comments below:

 * How does the output format used compare to other formats such as other popular formats such as BIDS (Brain Imaging Data Structure, which is maybe less general since it's intended for brain imaging data)?

 * Figure 2b (and the corresponding text): Raw saved running time, even though useful, is irrelevant without knowing the total running time. Please provide also the saved time as a percentage of the total running time.

 * Figures 2c and 2d: "1 lines" => "1 line"

 * The authors should consider renaming the "master" branch as the "main" branch (see https://sfconservancy.org/news/2020/jun/23/gitbranchname/).

 * Is there a reason for Python 3.8 not being tested in the continuous integration (CI) (maybe a limit on the number of jobs)? By the way, Python 3.7 is no longer supported and Python 3.11 has been released for over 9 months (https://devguide.python.org/versions/). The Python versions tested in the CI should probably be updated.

 * Flake8 is installed in the CI but never used. I ran the command locally (flake8 | wc -l) and obtained 2163 errors or warnings, which is not good. It would be nice to clean the source code a bit.

 * There is no code coverage associated to the tests, which is an important limitation. Please add code coverage to your project. There are "only" 16 tests for the moment, so I suspect that a substantial part of the source code is not tested.

 * I don't understand the point of using the "-s" option with pytest. I don't find the output (when using this option) to be clear or useful to identify tests that would fail for instance. In particular, I don't think that printing stuff when running the tests in the CI is relevant.

Are the conclusions about the tool and its performance adequately supported by the findings presented in the article?

Yes

Is the rationale for developing the new software tool clearly explained?

Yes

Is the description of the software tool technically sound?

Yes

Are sufficient details of the code, methods and analysis (if applicable) provided to allow replication of the software development and its use by others?

Yes

Is sufficient information provided to allow interpretation of the expected output datasets and any results generated using the tool?

Yes

Reviewer Expertise:

Open source software, Python programming, machine learning, medical applications

I confirm that I have read this submission and believe that I have an appropriate level of expertise to confirm that it is of an acceptable scientific standard, however I have significant reservations, as outlined above.

# Response

**Published:** 2024-04-05

**Authors:** Benjamin Haibe-Kains

**Affiliations:** Princess Margaret Cancer Centre, Canada

We thank Dr Faouzi for his feedback regarding our manuscript titled ‘Med-ImageTools: An open-source Python package for robust data processing pipelines and curating medical imaging data’. We have added our responses to their comments here in subdivided sections of comments/questions (Q), in bold, our corresponding responses/answers (A), and any relevant sections of the manuscript as an italicized bullet point, with changes emphasized in bold. 

 Q: The authors present their Python package for data processing pipelines and curating medical imaging data in this manuscript. Overall the manuscript is well written and clearly presents the main contributions of this package to the community. Nonetheless, some points on the software side could be improved. Please find my comments below:

**A: We thank the reviewer for their positive understanding of the authors’ work and constructive feedback, especially on the technical aspects of the package.**

 Q: How does the output format used compare to other formats such as other popular formats such as BIDS (Brain Imaging Data Structure, which is maybe less general since it's intended for brain imaging data)?

**A: We thank the reviewer for bringing the BIDS standard to the authors’ attention. The BIDS standard is very effective in standardizing brain imaging datasets by adhering to an intuitive structure describing various modalities of brain MRI. Med-ImageTools is mainly geared towards use cases that are broader and less specific than BIDS and its implementation would require fundamental reworkings of the package. However, the benefit of having a BIDS-native output cannot be understated and this feature will be added to the development team’s ongoing development goals. We have revised the relevant section from ‘Current Workflows’ as follows:**

- *While Med-ImageTools has many modular functions for image, contour, and dose input/output (IO) built on popular frameworks such as SimpleITK, TorchIO and PyDicom, such functionalities are redundant and available in other open-source packages as well. **We have tailored our core features towards broader use cases and development workflows, instead of modality or disease type specific workflows, such as BIDS. **Our main contribution is in the development of AutoPipeline and will mainly discuss its functionalities and implementation. *

Q: Figure 2b (and the corresponding text): Raw saved running time, even though useful, is irrelevant without knowing the total running time. Please provide also the saved time as a percentage of the total running time.

**A: We appreciate the reviewer’s point about Figure 2b. However, the reported time saved is reported in core-hours, which is a comprehensive unit of total running time. This is because the crawling occurred independently from the end-to-end processing of the datasets to ensure an accurate measure of core-hours saved. Reporting core-hours saved allows the reader to estimate their time/cost savings based on their specific hardware availabilities, either local machine, server-based, or cloud infrastructure, agnOSTIC to the hardware used by the authors. We have emphasIZED this point in our manuscRIPT as fOLLOWS:**

- *However, when scaled up to larger TCIA datasets such as OPC-Radiomics 13 (n=606) and NLST 14 (n=26254), it can save researchers 24.7 core-hours and 1072 core-hours, respectively (Figure 2b). **The resources saved are reported in core-hours to allow a hardware-agnostic estimation of time and cost savings. **While it may vary depending on the research infrastructure utilized, these databases can result in significant savings in billing. *

Q: Figures 2c and 2d: "1 lines" => "1 line"

 Q: The authors should consider renaming the "master" branch as the "main" branch (see https://sfconservancy.org/news/2020/jun/23/gitbranchname/).

 Q: Is there a reason for Python 3.8 not being tested in the continuous integration (CI) (maybe a limit on the number of jobs)? By the way, Python 3.7 is no longer supported and Python 3.11 has been released for over 9 months (https://devguide.python.org/versions/). The Python versions tested in the CI should probably be updated.

**A: We thank the reviewer for their comments. The figures and repository have been updated to address these points.**

 Q: Flake8 is installed in the CI but never used. I ran the command locally (flake8 | wc -l) and obtained 2163 errors or warnings, which is not good. It would be nice to clean the source code a bit.

**A: We thank the reviewer for raising the concern about the source code’s sanitation. The authors have reviewed the flake8 output and incorporated it into our codebase. You can find all changes in the following commit: https://github.com/bhklab/med-imagetools/commit/a8c0c3be53b4c9b43da12603b222d261d417a18c.**

 Q: I don't understand the point of using the "-s" option with pytest. I don't find the output (when using this option) to be clear or useful to identify tests that would fail for instance. In particular, I don't think that printing stuff when running the tests in the CI is relevant.

**A: We thank the reviewer’s attention to detail in Med-ImageTools’ CI/CD framework. The -s option is enabled for verbose outputs from Med-ImageTools to be carried through to the standard output of the tests in CI. In the past we have had OS-specific issues that were elucidated through the -s flag, but have removed it for future releases.**

 Q: There is no code coverage associated to the tests, which is an important limitation. Please add code coverage to your project. There are "only" 16 tests for the moment, so I suspect that a substantial part of the source code is not tested.

**A: We thank the reviewer for highlighting the lack of code coverage association of these tests. We agree that this is a clear limitation of the Med-ImageTools’ development pipeline, and have significantly increased the number of unit tests and integrated them into our CI/CD process. Future development efforts will continue to add more tests to our pipeline, including a badge detailing code coverage on our README.**

# Reviewer report: Reviewer response for version 1

**DOI:** 10.5256/f1000research.139617.r189015

**Published:** 2023-10-17

**Authors:** Rachel Sparks

**Affiliations:** 1 King's College London, London, England, UK

I think the motivation and justification for why a light weight Python module to support data loading and preprocessing of radiotherapy DICOM formats was very well articulated and clear. And I applaud the authors on making such a useful tool available to the community.

 However, I have two major concerns that must be addressed prior to this article being acceptable for indexing.

 First there are no details as to the design of Med-Image-Tools this includes important information such as:

 (1) dependencies on other python packages

 (2) what functions or other tools are being leveraged within the tool and how these are arranged.

 (3) the structure of the outputs of this pipeline specifically the csv file corresponding to the 'Dataset Index', the imaging and non-imaging feature outputs. For instance for imaging data how are pre-operative scans and segmentations saved and named (is there a convention is this something the user must write)

 (4) While there is a reference to the tutorial about the processing pipeline I think a high level overview listing the possible options would be appropriate to help the reader understand what this tool can offer.

 My other concern is related to the use cases. While lines of code is an easy metric to report it is mostly meaningless, some lines of code may be boilerplate or easy to write while others may obviously be different. It is also easy to pad or alter depending on the level of skill of the programmer. I think reporting run time is far more meaningful to your argument and I would stick to these.

 Finally, specifically, for the RADCURE dataset, it is not clear to me why you are comparing run time on a single core versus run time on a 32 core machine. I suspect the reported run time for the comparison could be significantly reduced by using a very simple parallelization which should be achievable tools available in python. I would suggest a fairer comparison for this specific example, it wont detract from the best argument you have which is there you are presenting a nice package so that other developers and researchers do not have to spend time re-writing code. Highlighting where some time savings may be gained is in some ways a secondary advantage.

 Finally, as a minor note the statement that SlicerRT is not easily interfaced with python is not a fully justified statement. One can use the python interface in Slicer to run python scripts and even run from the command prompt by called s'licer.exe  --python-code'. However, I think you can modify this to make a true and equally motivated statement which is that SlicerRT requires one to install Slicer/SlicerRT and stay within the slicer ecosystem to process data. Your tool lacks such dependencies so can be a more light weight and does not require running python through another software system.

Are the conclusions about the tool and its performance adequately supported by the findings presented in the article?

Yes

Is the rationale for developing the new software tool clearly explained?

Yes

Is the description of the software tool technically sound?

No

Are sufficient details of the code, methods and analysis (if applicable) provided to allow replication of the software development and its use by others?

No

Is sufficient information provided to allow interpretation of the expected output datasets and any results generated using the tool?

No

Reviewer Expertise:

Software development; open source software; medical image computing

I confirm that I have read this submission and believe that I have an appropriate level of expertise to confirm that it is of an acceptable scientific standard, however I have significant reservations, as outlined above.

# Response

**Published:** 2024-04-05

**Authors:** Benjamin Haibe-Kains

**Affiliations:** Princess Margaret Cancer Centre, Canada

We thank Dr Sparks for her feedback regarding our manuscript titled ‘Med-ImageTools: An open-source Python package for robust data processing pipelines and curating medical imaging data’. We have added our responses to their comments here in subdivided sections of comments/questions (Q), in bold, our corresponding responses/answers (A), and any relevant sections of the manuscript as an italicized bullet point, with changes emphasized in bold. 

 Q: I think the motivation and justification for why a light weight Python module to support data loading and preprocessing of radiotherapy DICOM formats was very well articulated and clear. And I applaud the authors on making such a useful tool available to the community.

**A: We thank the reviewer for their recognition of Med-ImageTools’ contribution to the medical imaging community and positively evaluating the authors’ works.**

 Q: However, I have two major concerns that must be addressed prior to this article being acceptable for indexing.

 First there are no details as to the design of Med-Image-Tools this includes important information such as:

 (1) dependencies on other python packages

**A: We thank the reviewer for pointing out the need to communicate the technical dependencies of Med-ImageTools. We have updated our ‘AutoPipeline’ section as follows, to include information on how to find the list of package dependencies. **

- *Running the ‘ pip install med-imagetools’ command will install the latest version of Med-ImageTools and its associated dependencies **defined by the requirements.txt file.***

**The ‘Current workflows’ section also contains a concise description of what frameworks Med-ImageTools leverages such as follows:**

- *While Med-ImageTools has many modular functions for image, contour, and dose input/output (IO) built on popular frameworks such as SimpleITK, TorchIO and PyDicom…*

Q: (2) what functions or other tools are being leveraged within the tool and how these are arranged.

**A: We thank the reviewer for calling attention to the need to clarify how other tools/packages function within Med-ImageTools. We have added some information to our ‘AutoPipeline’ section to clarify major operations performed using external tools/packages as follows. For a granular understanding of the integration behind every external tool, we defer the reader to learn about the design choices made throughout the package and its implications by gaining a deeper understanding of the source code available through our GitHub repository. **

- *1. Crawl: The crawler opens every DICOM file in the dataset using Pydicom, indexing important metadata, such as unique identifiers, modality information, and references to other modalities. This produces a database of every unique image and DICOM-RT modality.*
- *3. Process: All identified samples are processed according to imaging and transformation parameters defined by the user. The user can configure parameters such as the pixel spacing in mm(s), which specific modalities to process, the number of cores they want to use for multiprocessing, and define nnU-Net specific flags as well. **The images are manipulated using SimpleITK without requiring user intervention. ***
- *As the crawl and process steps are computationally intensive, all steps in AutoPipeline are automatically parallelized **using the joblib backend** to efficiently leverage all available computational resources.*

Q: (3) the structure of the outputs of this pipeline specifically the csv file corresponding to the 'Dataset Index', the imaging and non-imaging feature outputs. For instance for imaging data how are pre-operative scans and segmentations saved and named (is there a convention is this something the user must write)

**A: We thank the reviewer for this question and for highlighting the lack of clarity of our description of AutoPipeline. The ‘Dataset Index’ is designed to be an autogenerated output of the Crawler and does not require any user interaction to be created; this is simply there to ensure convenient user experience. We have made this and other aspects of the outputs clearer by adding the following sentences to the ‘AutoPipeline’ section.**

- ***Med-ImageTools also generates files that are not analysis-ready images, such as the autogenerated ‘Dataset Index’ (Figure 1), and stores them in a folder named “.imgtools” at the path of the ‘input_directory’. This is to ensure a convenient user experience and intuitive folder structure by hiding extraneous components.***

Q: (4) While there is a reference to the tutorial about the processing pipeline I think a high level overview listing the possible options would be appropriate to help the reader understand what this tool can offer.

**A: We agree with the reviewer that adding this information to our description of AutoPipeline can help readers quickly understand the tool’s strengths before interfacing with the GitHub repository or ReadTheDocs. We have updated the ‘AutoPipeline’ section accordingly.**

- *3. Process: All identified samples are processed according to imaging and transformation parameters defined by the user. **The user can configure parameters such as: the pixel spacing in mm(s), which specific modalities to process, the number of cores they want to use for multiprocessing, and define nnU-Net specific flags as well. The images are manipulated using SimpleITK without requiring user intervention. ***

Q: My other concern is related to the use cases. While lines of code is an easy metric to report it is mostly meaningless, some lines of code may be boilerplate or easy to write while others may obviously be different. It is also easy to pad or alter depending on the level of skill of the programmer. I think reporting run time is far more meaningful to your argument and I would stick to these.

**A: We appreciate the reviewer’s consideration in the metrics reported in Figure 2. However, we believe this metric emphasizes Med-ImageTools’ strength allowing users to process DICOM datasets with a single command line interaction, in contrast to writing dozens of lines of Python/R/MATLAB scripts. One particular reason why we believe a single command line is useful to highlight is to express the ease-of-use for users that may not be comfortable writing their own code for DICOM to NifTI/Nrrd processing, especially if they lack deep domain knowledge.**

**Another, perhaps more, exciting reason is to show the potential of building automated workflows on top of Med-ImageTools. If one were to build an end-to-end automated pipeline starting from clinical DICOM datasets to outputting an inference-ready deep learning model, they could easily develop a reproducible processing step by configuring only the command line interaction of Med-ImageTools, making debugging and custom configurations simpler since the developer would not have to rely on a static script. We have elaborated on Med-ImageTools’ contributions for improved scientific reproducibility in the ‘Discussion’ section as follows. **

- *The AutoPipeline feature will improve the accessibility of raw clinical datasets on public archives, such as TCIA, allowing machine learning researchers to process analysis-ready formats without requiring deep domain knowledge. **Another exciting potential of Med-ImageTools lies in building automated workflows using AutoPipeline. For a researcher to build an end-to-end automated pipeline starting from clinical DICOM datasets to outputting an inference-ready deep learning model, they could easily develop a reproducible processing step by configuring only the command line interaction of Med-ImageTools, making debugging and custom configurations simpler since the developer would not have to rely on a static script. ***
- *No single solution can completely solve the reproducibility crisis of medical deep learning research, due to a variety of issues ranging from ambiguous data processing techniques to stochasticity of model training. However, community-centered open-source solutions and increased clinical adherence to data standards, such as contour nomenclature, 15 can incrementally improve research quality and reproducibility, and make medical deep learning research more accessible for everyone.*

Q: Finally, specifically, for the RADCURE dataset, it is not clear to me why you are comparing run time on a single core versus run time on a 32 core machine. I suspect the reported run time for the comparison could be significantly reduced by using a very simple parallelization which should be achievable tools available in python. I would suggest a fairer comparison for this specific example, it wont detract from the best argument you have which is there you are presenting a nice package so that other developers and researchers do not have to spend time re-writing code. Highlighting where some time savings may be gained is in some ways a secondary advantage.

**A: We thank the reviewer for their comment about Figure 2d. The goal of the comparison was not only to show a reduction in processing time based on parallelization, but to show that our package’s design does not bottleneck any multiprocessing backends and brings meaningful acceleration on large datasets. In other words, we wanted to show that a 32x increase in computational hardware was going to bring at least 32x improvement in performance as observed in Figure 2d. This is in contrast to Figure 2c which does not show similar levels hardware acceleration, but its usefulness in parsing private, unseen data. We have expanded on these ideas in the manuscript as follows:**

- *In contrast, on the same 6-core machine (16GB RAM, Windows 10), AutoPipeline took only 2.3 hours with 1 command line, mainly accelerated by parallelization ( Figure 2c). **The authors involved in validating Med-ImageTools' effectiveness on this private dataset had no active involvement in the development of the package before its application. This highlights the package’s robustness on unseen data and its potential utility for multi-centre collaborations to ensure consistent processing. One use case might be to enable federated learning platforms to automatically process each node’s datasets without requiring any user intervention.***
- *AutoPipeline scales dramatically based on the number of cores available, which enables the entire dataset to be processed in 40 minutes using 32 cores, automatically managing the directories from different systems and extracting the contours ( Figure 2d). **These results demonstrate Med-ImageTools' design does not bottleneck any multiprocessing backends and brings meaningful acceleration on very large datasets, such as RADCURE. ***

Q: Finally, as a minor note the statement that SlicerRT is not easily interfaced with python is not a fully justified statement. One can use the python interface in Slicer to run python scripts and even run from the command prompt by called s'licer.exe  --python-code'. However, I think you can modify this to make a true and equally motivated statement which is that SlicerRT requires one to install Slicer/SlicerRT and stay within the slicer ecosystem to process data. Your tool lacks such dependencies so can be a more light weight and does not require running python through another software system.

**A: We thank the reviewer for this point. We have revised the relevant section from ‘Current Workflows’ as follows to better justify our statement.**

- *Despite its broad adoption, **batch data processing with Slicer requires custom scripting in Python, to be executed in the Slicer ecosystem. Rather than simply installing a package within their Python environment, users must install the Slicer application and add any other dependencies, not provided by Slicer, into the application environment. As a result, machine learning projects relying on Python for data preprocessing will have their code fragmented across multiple environments–the Slicer environment for data processing, and another Python environment for data analysis and machine learning.***
